# Radiographic and clinical evaluation of anterior nasal spine anatomy on anterior maxillary implant placement

**DOI:** 10.6026/973206300212820

**Published:** 2025-08-31

**Authors:** Swapnil Uttamrao Shinde, Pradeep Kumar Sharma, Manoj Upadhyay, Suman Thotapalli, Munaz Mulla, Sajith Abraham, Subham Patra, Mushir Mulla

**Affiliations:** 1 Department of Oral and Maxillofacial Surgery, Bharati Vidyapeeth (Deemed to be University) Dental College and Hospital, Sangli, Maharashtra, India; 2 Department of Oral and Maxillofacial Surgery, Government Dental College, Dibrugarh, Assam, India; 3 Department of Dentistry, Government Medical College, Barmer, Rajasthan, India; 4 Department of Prosthodontics, SB Patil Institute of Dental Sciences and Research, Bidar, Karnataka, India; 5 Department of Periodontics, Saveetha Institute of Dental College and Hospitals, Saveetha Institute of Medical and Technical Sciences, Saveetha University, Chennai, Tamilnadu, India; 6 Department of Preventive Dental Sciences, College of Dentistry, King Faisal University, AlHassa, Saudi Arabia; 7 Intern, Kalinga Institute of Dental Science, Kalinga Institute of Industrial Technology (KIIT) Deemed to be University, Patia, Bhubaneswar, Odisha, India; 8 Department of Public Health, College of Applied Medical Sciences, Qassim University, Saudi Arabia

**Keywords:** Anterior nasal spine, CBCT, anterior maxilla, implant planning, alveolar bone

## Abstract

The anterior nasal spine (ANS) is an important anatomical landmark that can influence on anterior implant placement. Therefore, it is of interest to evaluate ANS morphology (radiographic and clinical) and its influence on planning of anterior implants placement. Hencec, 100 adult subjects were divided into Group A with favorable ANS morphology; Group B with unfavorable ANS morphology). Cone-beam CT (CBCT) measurements included ANS length (mm), ANS angulation (°), labial cortical thickness, available alveolar bone height and width at planned implant sites. Clinical assessment recorded ridge contour and palpation findings. ANS anatomy significantly correlates with anterior maxillary bone availability and influences immediate implant planning.

## Background:

The anterior nasal spine (ANS) is a midline bony projection located at the inferior border of the piriform aperture, contributing to nasal support, premaxillary contour and dentofacial harmony. Its anatomical morphology-including length, angulation and proximity to the incisive foramen-can have a profound influence on anterior maxillary implant planning by affecting labial bone thickness, alveolar bone height and available axial bone trajectory [1]. Unfavourable ANS morphology may increase the risk of labial plate perforation, compromise esthetics, or necessitate augmentation procedures [2]. Accurate preoperative assessment of the ANS and surrounding anatomy has become increasingly feasible with cone-beam computed tomography (CBCT), which allows precise three-dimensional evaluation of bone quantity, angulation and the relationship to neighboring structures [3]. CBCT-based morphometric studies have demonstrated considerable variation in the anterior maxillary region, with additional attention drawn to anatomical channels and nasopalatine canal morphology that may influence implant positioning [4]. Furthermore, longitudinal imaging studies have shown that bone dimensions in this region can change over time post-implantation, underscoring the importance of preoperative anatomical mapping using reproducible landmarks such as the ANS [5]. Despite advancements in surgical protocols and augmentation techniques for anterior maxillary implants [6], current literature provides limited focused investigation into the specific role of ANS morphology in determining implant feasibility. Most prior research has emphasized general anatomical factors [7] or addressed the ANS in contexts unrelated to implant planning-such as bone harvesting for grafting [8], maxillofacial landmark identification [9], or orthodontic mini-implant placement in the ANS region [10]. No comprehensive cross-sectional study, to our knowledge, has combined both clinical palpation and radiographic measurements of ANS morphology to directly assess its predictive value for implant feasibility without additional augmentation. Therefore, it is of interest to describe the gap by systematically evaluating ANS anatomy via CBCT and correlating these findings with clinical feasibility assessments for anterior maxillary implant placement.

## Materials and Methods:

This cross-sectional observational analytic study was conducted following approval from the Institutional Ethics Committee. Written informed consent was obtained from all participants prior to inclusion. The total sample comprised 200 adult participants aged 18-65 years. For clear between-group contrasts, subjects were allocated into two equal groups: Group A (n = 100) included participants with favorable ANS morphology and Group B (n = 100) included those with unfavorable ANS morphology. Favorable morphology was defined radiographically as ANS length ≥ 5.8 mm, ANS angulation within ± 10° of a normative reference line (based on pilot data) and labial bone thickness ≥ 1.5 mm at the crest-parameters expected to favor implant placement without grafting. Unfavorable morphology was defined as ANS length < 5.8 mm and/or pronounced retro- or antero-angulation and labial bone thickness < 1.5 mm. Inclusion criteria were adults aged 18-65 years who required CBCT imaging for diagnostic or implant planning purposes in the anterior maxilla, or who presented for dental care with an existing CBCT and who had at least one site in the central incisor-to-canine region considered for implant planning. Exclusion criteria included a history of trauma or surgery involving the ANS or anterior nasal complex, presence of major craniofacial syndromes, active infection in the anterior maxilla and pregnancy. All CBCT scans were obtained using a standardized protocol (voxel size 0.2-0.3 mm; field of view covering the maxilla) and assessed in multiplanar reconstructions (axial, coronaland sagittal) using dedicated workstation software. Radiographic measurements included ANS length (horizontal projection from the anterior edge of the piriform rim to the ANS tip, in mm), ANS angulation (degrees relative to the maxillary plane), labial cortical thickness at the crest and 3 mm apical, available alveolar bone height from the crest to the nasal floor/incisive canal, alveolar width at 3 mm and 6 mm from the crest and distance to the incisive foramen. Each measurement was performed twice by two calibrated observers, with intra- and inter-examiner intraclass correlation coefficients (ICC) calculated. Clinical assessments included palpation of the ANS (prominent vs flat), ridge contour classification (Class I-III, adapted from the alveolar ridge classification) and soft tissue biotype (thin vs thick). Implant feasibility without grafting was the primary outcome, categorized as feasible, borderline (potentially requiring minor augmentation or a narrower implant), or not feasible (requiring significant augmentation or alternative planning). Two experienced implant surgeons, blinded to group assignment, independently assessed each case, resolving any disagreements by consensus. Statistical analysis involved testing for normality with the Shapiro-Wilk test. Continuous variables were expressed as mean ± SD and compared using the independent t-test (Welch's correction applied when variances were unequal). Categorical variables were presented as counts and percentages and compared using the chi-square test. Logistic regression was performed to assess ANS length (per 1 mm increment) as a predictor of implant feasibility, adjusting for age, sex and ridge width. Statistical significance was set at p < 0.05. Analyses were conducted using SPSS version 23 (IBM Corp., Armonk, NY) software.

## Results:

The study included a total of 200 participants, with 100 individuals in each group (Group A and Group B). The mean age was 41.2 ± 11.3 years in Group A and 43.0 ± 10.8 years in Group B, showing no statistically significant difference (p = 0.27). The sex distribution was comparable between groups, with females comprising 54% of Group A and 50% of Group B (p = 0.58). Table 1(see PDF) summarizes key CBCT findings, showing that Group A had significantly greater ANS length, available bone height and labial cortical thickness at the crest and alveolar width at 3 mm compared to Group B (all p < 0.001). In the present study, implant placement feasibility in the anterior maxilla was assessed in relation to the anatomical characteristics of the anterior nasal spine (ANS). In Group A (favorable ANS morphology), the majority of sites were categorized as feasible for implant placement (86%), followed by borderline cases (10%) and a small proportion deemed not feasible (4%). In contrast, Group B (unfavorable ANS morphology) showed a markedly lower proportion of feasible sites (42%), with higher rates of borderline (28%) and not feasible (30%) cases. The chi-square test revealed a statistically significant association between ANS morphology group and implant placement feasibility (χ^2^ = 43.53, df = 2, p < 0.001), indicating that favorable ANS anatomy is strongly correlated with improved implant feasibility in the anterior maxilla Table 2(see PDF). In the logistic regression analysis, implant feasibility (yes/no) was modeled as the binary outcome, with anterior nasal spine (ANS) length as the primary predictor, adjusting for age, sex and crest width. The adjusted odds ratio (OR) was 1.45 per 1 mm increase in ANS length (95% CI: 1.25-1.68, p < 0.001), indicating that each additional millimeter in ANS length was associated with a 45% higher likelihood of achieving implant feasibility after controlling for the covariates. This suggests that ANS length is a strong anatomical determinant in the simulated scenario. Reliability testing demonstrate excellent agreement between examiners, with an intra-class correlation coefficient (ICC) of 0.92 for linear measurements and a Cohen's kappa value of 0.84 for categorical feasibility assessments, reflecting high consistency in both quantitative and qualitative evaluations (Table 3 - see PDF).

## Discussion:

The anterior nasal spine (ANS) serves as a key maxillofacial landmark, exerting a notable influence on the morphology of the premaxillary region and thereby affecting both the quantity and quality of bone available for anterior maxillary implant placement. Its anatomical variations-such as length, angulation and cortical thickness-can alter the local bone architecture, impacting implant site preparation, primary stability and the need for augmentation procedures. Recent morphometric research has highlighted the clinical importance of ANS assessment, emphasizing its role in pre-surgical planning and orthodontic preparation, particularly in optimizing upper incisor positioning and evaluating skeletal support in the premaxilla [3]. Additionally, cone-beam computed tomography (CBCT) studies have shown that detailed visualization of the ANS region not only aids in implant planning but also assists in identifying adjacent anatomical structures, including accessory bone channels, which may influence surgical access and risk management [4]. Together, these insights underscore the need for a comprehensive radiographic and clinical evaluation of ANS morphology as an integral part of anterior maxillary implant planning. The demographic characteristics of our sample showed no statistically significant differences in age or sex distribution between the two groups, ensuring comparability and minimizing potential confounding effects. The mean age values in both groups fall within the adult range typically represented in CBCT-based maxillofacial evaluations, consistent with prior anatomical studies of the anterior nasal spine region [9]. Similarly, the balanced sex distribution aligns with previous CBCT-based morphometric research, where no strong sex-related differences in ANS-related implant planning feasibility were observed [10]. This demographic uniformity strengthens the validity of subsequent radiographic and clinical comparisons by reducing demographic bias. The possibility of dental implants penetrating the nasal cavity restricts their placement in the highly resorbed anterior maxillary alveolar ridge [11]. In order to address peri-implant bone deficiencies, especially when implanting in the anterior maxilla, the anterior nasal spine (ANS) is commonly utilised [12]. In the present study, CBCT analysis revealed significantly greater ANS length, available bone height, labial cortical thickness and alveolar width at 3 mm in Group A compared to Group B, with all differences being statistically significant (p < 0.001). These findings highlight the morphological advantage conferred by a longer ANS in terms of implant site quality, as greater bone height and cortical support are critical for achieving primary stability and long-term success in anterior maxillary implants [1, 2].

The positive correlation between ANS prominence and labial cortical thickness aligns with previous morphometric evaluations, which have reported that premaxillary bone morphology is closely influenced by ANS anatomy [3, 7]. From a surgical perspective, enhanced labial cortical thickness and alveolar width in the favorable group may reduce the need for augmentation procedures; consistent with literature noting that adequate buccolingual dimension at the crest is essential for optimal implant biomechanics [5, 6]. Furthermore, the increased vertical bone availability observed in Group A is particularly relevant for aesthetic zone rehabilitation, where both bone volume and contour maintenance are critical for soft tissue support [2, 6]. These results reinforce the role of detailed CBCT assessment in preoperative planning, not only for implant positioning but also for anticipating potential anatomical constraints and optimizing surgical strategies [4, 8]. The present findings demonstrate a robust association between favorable ANS morphology and increased feasibility of anterior maxillary implant placement. Group A, characterized by greater ANS prominence and length, exhibited more than double the proportion of feasible sites compared to Group B, highlighting the role of ANS-related morphology in providing adequate bone volume and favorable angulation for implant anchorage. Logistic regression further confirmed that ANS length was a significant independent predictor, with each additional millimeter associated with a 45% increase in the odds of implant feasibility, even after adjusting for demographic and ridge width variables. This aligns with prior CBCT-based morphometric studies, which reported that a well-developed ANS contributes to improved alveolar bone height and labial plate thickness in the premaxillary region, reducing the need for advanced grafting procedures [2, 5 and 7]. The high inter-examiner agreement underscores the reproducibility of these CBCT-derived anatomical assessments, consistent with previous reliability reports in anterior maxillary measurements [4, 6]. From a clinical standpoint, these results emphasize the importance of incorporating ANS morphology into routine implant site evaluation, as it may influence both surgical planning and long-term esthetic outcomes, especially in the highly visible anterior zone [3, 6]. In conclusion, a precise understanding of anterior nasal spine anatomy emerges as a pivotal guidepost in optimizing anterior maxillary implant placement, harmonizing surgical precision with predictable esthetic and functional outcomes.

## Limitation:

The study's findings may be limited by the sample size and the single-center design, which could affect generalizability.

## Future perspective:

Multi-center studies with larger and more diverse populations are recommended to validate and expand on these results.

## Conclusion:

ANS anatomy is a meaningful predictor of anterior maxillary bone availability and the feasibility of implant placement. Incorporating ANS measurement into radiographic implant planning can improve treatment predictability and inform decisions on grafting, implant diameter and angulation.
